# Genotyping of Genetically Monomorphic Bacteria: DNA Sequencing in *Mycobacterium tuberculosis* Highlights the Limitations of Current Methodologies

**DOI:** 10.1371/journal.pone.0007815

**Published:** 2009-11-12

**Authors:** Iñaki Comas, Susanne Homolka, Stefan Niemann, Sebastien Gagneux

**Affiliations:** 1 Division of Mycobacterial Research, Medical Research Council, National Institute for Medical Research, London, United Kingdom; 2 Molecular Mycobacteriology, Research Center Borstel, Borstel, Germany; Duke University Medical Center, United States of America

## Abstract

Because genetically monomorphic bacterial pathogens harbour little DNA sequence diversity, most current genotyping techniques used to study the epidemiology of these organisms are based on mobile or repetitive genetic elements. Molecular markers commonly used in these bacteria include Clustered Regulatory Short Palindromic Repeats (CRISPR) and Variable Number Tandem Repeats (VNTR). These methods are also increasingly being applied to phylogenetic and population genetic studies. Using the *Mycobacterium tuberculosis* complex (MTBC) as a model, we evaluated the phylogenetic accuracy of CRISPR- and VNTR-based genotyping, which in MTBC are known as spoligotyping and Mycobacterial Interspersed Repetitive Units (MIRU)-VNTR-typing, respectively. We used as a gold standard the complete DNA sequences of 89 coding genes from a global strain collection. Our results showed that phylogenetic trees derived from these multilocus sequence data were highly congruent and statistically robust, irrespective of the phylogenetic methods used. By contrast, corresponding phylogenies inferred from spoligotyping or 15-loci-MIRU-VNTR were incongruent with respect to the sequence-based trees. Although 24-loci-MIRU-VNTR performed better, it was still unable to detect all strain lineages. The DNA sequence data showed virtually no homoplasy, but the opposite was true for spoligotyping and MIRU-VNTR, which was consistent with high rates of convergent evolution and the low statistical support obtained for phylogenetic groupings defined by these markers. Our results also revealed that the discriminatory power of the standard 24 MIRU-VNTR loci varied by strain lineage. Taken together, our findings suggest strain lineages in MTBC should be defined based on phylogenetically robust markers such as single nucleotide polymorphisms or large sequence polymorphisms, and that for epidemiological purposes, MIRU-VNTR loci should be used in a lineage-dependent manner. Our findings have implications for strain typing in other genetically monomorphic bacteria.

## Introduction

Some of the most important bacterial pathogens of humans exhibit strikingly low DNA sequence diversity. On average, these organisms harbour one nucleotide difference every 2–28 k base pairs and are thus referred to as genetically monomorphic [1]. Some prominent examples include *Yersinia pestis* (the etiologic agent of plague) [2], *Salmonella enterica* serovar Typhi (typhoid fever) [3], *Bacillus anthracis* (anthrax) [4], as well as the three most important pathogenic mycobacteria, *Mycobacterium leprae* (leprosy) [5], *Mycobacterium ulcerans* (buruli ulcer) [6], and *Mycobacterium tuberculosis* complex (MTBC) [7]. MTBC includes several sub-species that cause tuberculosis in humans and in various other mammals.

**Figure 1:**
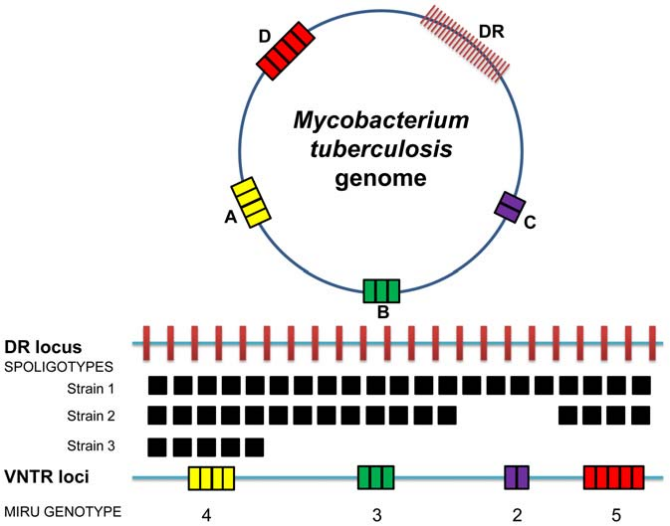
Schematic illustrating the principles of the CRISPR- and VNTR-based genotyping in MTBC. These genotyping methods are known as ‘spoligotyping’ and ‘MIRU-VNTR-typing’, respectively. Spoligotyping is based on the detection of 43 unique spacers located between direct repeats at a specific locus of the MTBC genome known as the direct repeat (DR) locus. Spoligotyping patterns are commonly represented by black and white squares indicating presence or absence of particular spacers, respectively. The deletion of some of these 43 spacers allows to differentiate between strains. MIRU-VNTR analysis relies on the identification of different number of repeats at several loci scattered around the bacterial genome (marked by A, B, C, and D in the figure). The number of repeats at each locus is combined to generate a unique numerical code used to establish phylogenetic and epidemiological links between strains.

Understanding the diversity of bacterial pathogens is important, both for epidemiological and biological reasons. However, because of the low DNA sequence variation in monomorphic bacteria, studying the genetic diversity of these microbes is challenging. Standard sequence-based methods like multilocus sequence typing (MLST) are not applicable because of low phylogenetic resolution [8], [9]. Alternative non-sequence-based tools, such as Pulsfield Gel Electrophoresis (PFGE) and Restriction Fragment Length Phylomorphism (RFLP) have been used for fine typing of monomorphic bacteria. However, these gel-based techniques have many draw-backs and are difficult to reproduce within and between laboratories [1]. More recently, PCR-based genotyping methods have been developed. Two of the most popular techniques are based on Clustered Regulatory Short Palindromic Repeats (CRISPR) and Variable Number Tandem Repeats (VNTR), respectively (Figure 1) [10], [11]. CRISPRs are regions of the bacterial genome characterized by series of direct repeats interspersed by short unique regions called spacers. CRISPRs have been shown to encode a specialized defence mechanisms against bacteriophages, and changes in the number of spacers have been associated with phage-susceptibility [12]. VNTR-typing on the other hand, compares the strain-specific numbers of repeats of short DNA sequences at different positions of the bacterial genome [11].

CRISPR- and VNTR-based genotyping has been established for many genetically monomorphic bacterial pathogens, including *Y. pestis* [13], [14], [15], *B. anthracis* [16], *Salmonella eneterica* serovar Typhi [17], *Francisella tuleransis* [18], *Escherichia coli* O157 [19], and *M. leprae* [20]. In MTBC, the corresponding CRISPR- and VNTR-based methodologies are known as spoligotyping and MIRU-VNTR, respectively (Figure 1) [21], [22]. These two genotyping techniques were originally developed for molecular epidemiological applications, and are routinely used to trace ongoing chains of tuberculosis transmission [23], to differentiate cases of disease relapse from re-infections [24], and to detect laboratory cross-contamination [25]. Over the years, databases have been populated with spoligotyping and MIRU-VNTR-typing results from thousands of patient isolates. For example, the spoligotyping database SpolDB4 contains data from close to 40,000 MTBC isolates from more than 120 countries [26], and MIRU-VNTR*plus* has been put up as a new online reference database for standard genotyping of MTBC [27].

In addition to routine molecular epidemiological applications, spoligotyping and MIRU-VNTR are increasingly also being applied to study evolutionary questions. Two complementary sets of MIRU-VNTR loci have been developed for MTBC [28]; 15-loci-MIRU-VNTR, which includes 15 loci, originally found to be the most discriminatory, and 24-loci-MIRU-VNTR that includes the same 15 loci plus an additional nine, which provide additional phylogenetic information. While 15-loci-MIRU-VNTR is mainly being applied for routine molecular epidemiology, spoligotyping and 24-loci-MIRU-VNTR have been proposed for phylogenetic and population genetic analyses of MTBC [26], [29], [30].

We recently performed a multilocus sequence analysis (MLSA) of 108 MTBC strains in which we generated the complete coding sequences of 89 genes, corresponding to ∼70 k base pairs per strain [31]. We used these DNA sequences to generate a highly robust phylogeny of MTBC [31], [32]. Here we used this MLSA-based phylogeny to evaluate the phylogenetic accuracy of spoligotyping and MIRU-VNTR in MTBC. In addition, we used this MLSA dataset to investigate the discriminatory power of the 24 standard MIRU-VNTR loci in the different strain lineages of MTBC.

## Results

### DNA Sequencing Defines a New Gold-Standard for the Phylogenetic Classification of MTBC

**Figure 2:**
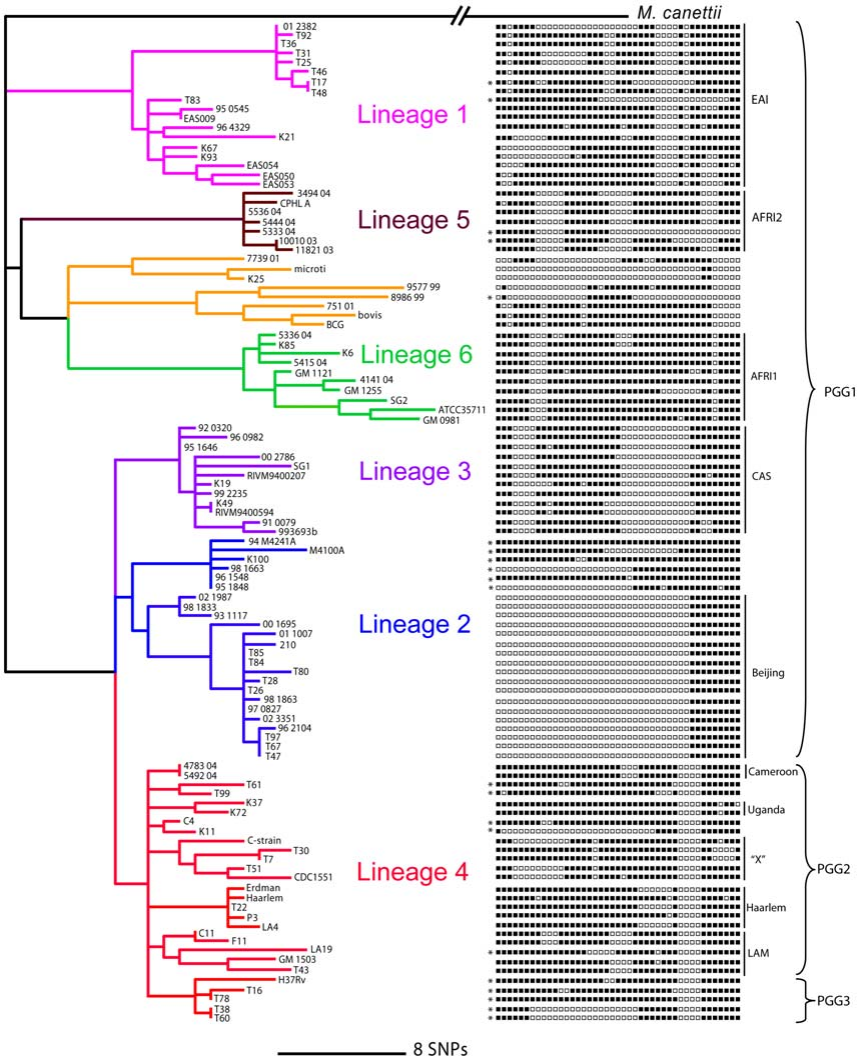
Maximum parsimony phylogeny based on concatenates of 89 gene sequences from 108 MTBC strains from global sources as previously reported [**31**] **.** Six main lineages can be observed within the human MTBC (numbered 1 to 6 and indicated in different colours). As shown previously, these lineages are highly congruent to the ones defined based on genomic deletions or large sequence polymorphisms (LSPs) [31], [33], [34]. Corresponding spoligotyping data for each strain are shown on the right, where black squares indicate the presence of a particular spacer and a white square the absence of a particular spacer (see Figure 1 for details on the methodology). Because the various typing techniques have classified MTBC strains into several lineages and strain families using differing nomenclatures, some of the traditional names are also shown. Some of the traditional groupings defined by spoligotyping correlate with SNP-based lineages (see also Table S1). For example, EAI (East-African-Indian) corresponds to the pink lineage, AFR1 and AFR2 correspond to the green and brown lineage, respectively (these strains are also known as *M. africanum*), and CAS (Central-Asian) corresponds to the purple lineage. However, other strain groupings defined by spoligotyping should be regarded as sub-lineages within the main lineages. For example, the ‘Beijing’ strain family is part of the blue lineage, and the five spoligotyping groups ‘Cameroon’, ‘Uganda’ ‘X’, ‘Haarlem’, and ‘LAM (Latin-American-Mediterranean)’ are sub-lineages within the main red lineage. This highlights another limitation of spoligotyping, which is that phylogenetic relationships between strain groupings cannot be defined. In addition, asterisks indicate spoligotyping patterns that cannot be classified at all using standard ‘signature patterns’ [26]. PGG1, PGG2, and PGG3 indicate Principal Genetic Group 1, 2, and 3, respectively. The PGG nomenclature is based on two SNPs originally described by Sreevatsan at al. [7]. Comparison to the MLSA data shows these groups are not phylogenetically equivalent as most of the MTBC diversity groups within PGG1, and PGG3 includes only a small subset of strains.

**Figure 3:**
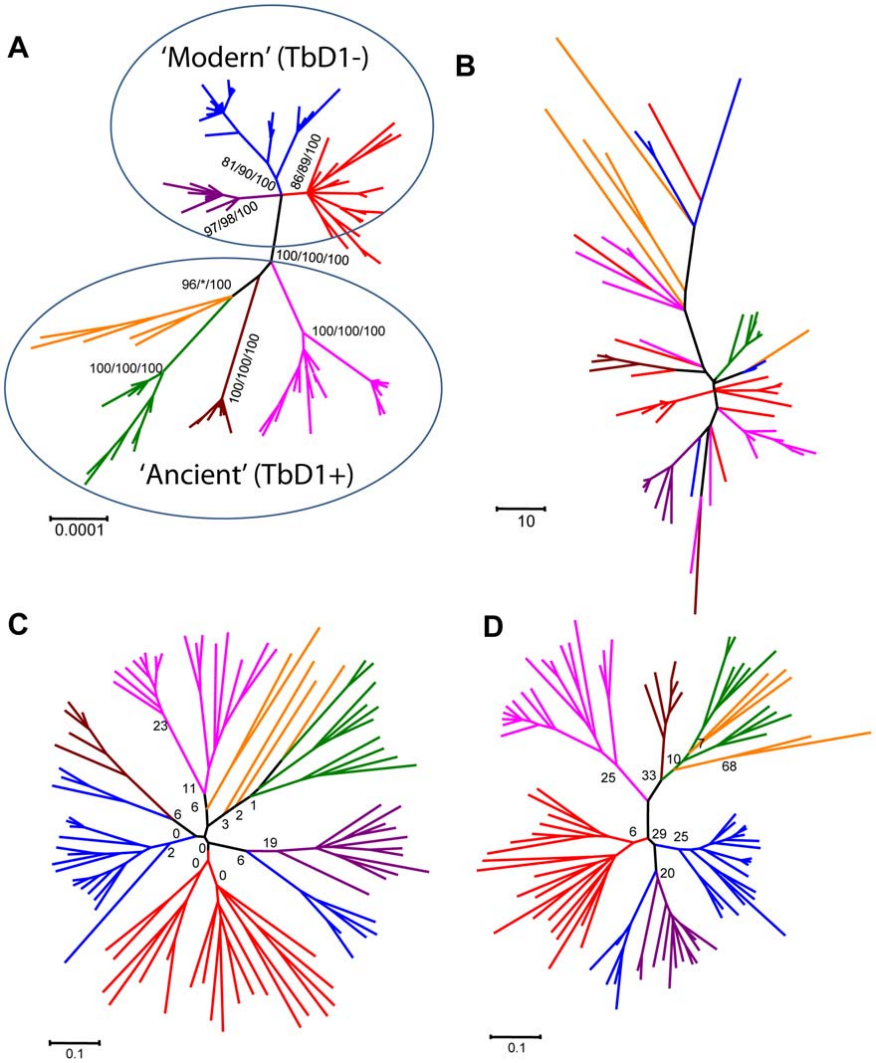
Comparison of unrooted phylogenies of MTBC based on 97 global strains using various molecular markers. Colours indicate the main MTBC lineages as defined by MLSA and LSPs [31]. (**A**) Neigbour-joining (NJ) phylogeny based on 339 variable nucleotide positions in 89 genes using number of SNPs as distance. The same topology was obtained using NJ, Maximum likelihood (ML), and Bayesian inference (BI). Numbers indicate bootstrap support after 1,000 pseudoreplicates for NJ and ML, and *a posteriori* probabilities for BI, respectively (Figure S1). MTBC can be divided in two main clades, one evolutionary ‘modern’ (also known as ‘TbD1-negative’), which includes the blue, purple, and red strain lineages, and one evolutionary ‘ancient’ (TbD-positive), which includes the remaining strain lineages. (**B**) NJ phylogeny based on spoligotyping data and Jaccard distances. No bootstrap values could be calculated using these markers. (**C**) NJ phylogeny based on 15-loci-MIRU-VNTR data and Nei distances. Numbers indicate bootstrap support after 1,000 pseudoreplicates. (**D**) NJ phylogeny based on 24-loci-MIRU-VNTR data and Nei distances. Numbers indicate bootstrap support after 1,000 pseudoreplicates.

We previously showed that MLSA of 108 global strains of MTBC resulted in a single most parsimonious phylogenetic tree with negligible homoplasy (Figure 2) [31]. This phylogeny was also highly congruent with our previous analyses based on large sequence polymorphisms (LSPs) [33], [34], and earlier DNA sequencing work [8], [35]. To further probe the robustness of our MLSA-based phylogeny, we re-analyzed our DNA sequence data by the Neighbour-joining, Maximum likelihood, and Bayesian inference methods. All three analyses yielded identical tree topologies, which were highly congruent with our previous findings based on Maximum parsimony (Figure 2, Figure 3A, Figure S1). Furthermore, high statistical support was obtained for all main clades and for each method, despite the fact that some lineages were defined by only a small number of single nucleotide polymorphisms (SNPs). Because MTBC is strictly clonal [36], [37], and our LSP and MLSA analyses were highly congruent, we conclude that our MLSA-based phylogeny is robust and appropriate for classification of MTBC strains into discrete strain lineages.

To develop a new SNP-based classification system for MTBC, we used our MLSA-based phylogeny to extracted all lineage-defining SNPs (Table S1). Many of these SNPs are redundant and can be used interchangeably to identify the same phylogenetic groupings. Because the technical requirements of various SNP-typing technologies may vary [38], [39], we believe being able to choose among more than one lineage-specific SNP will facilitate the design of SNP-based assays using either of these platforms. Furthermore, the SNPs proposed here are more appropriate for typing of MTBC compared to most of the ones reported previously, because we used *de novo* DNA sequence data from 108 global strains to discover these SNPs [31]. By contrast, SNP collections published previously were identified by comparing only three or four MTBC genome sequences [40], [41], [42], and thus suffer from phylogenetic discovery bias [1], [32], [43], [44], [45].

### CRISPR- and VNTR-Based Genotyping Results in Unreliable Phylogenetic Inference

CRISPR- and VNTR-based genotyping techniques have been used for phylogenetic and population genetic studies of genetically monomorphic bacteria [1]. Here we evaluated the performance of some of these methods using MTBC as an example. We used both qualitative and quantitative methods to determine the phylogenetic congruence of CRISPR-based spoligotyping and MIRU-VNTR-typing using our MLSA-based phylogeny as a gold standard.

We first generated the corresponding spoligotyping and 24-loci-MIRU-VNTR-typing data from strains included in our MLSA study following internationally standardized protocols [21], [28]. Our final dataset comprised 97 strains with complete MLSA, spoligotyping, 15-loci-MIRU-VNTR, and 24-loci-MIRU-VNTR data (Table S2). We then used a qualitative approach to see whether the main MTBC lineages inferred by MLSA (indicated in different colours in Figure 2 and Figure 3A) were reproduced across the different genotyping datasets. To test this, we mapped the seven main MTBC lineages onto the tree topologies generated from the spoligotyping, 15-loci-MIRU-VNTR, or 24-loci-MIRU-VNTR data (Figures 3B, 3C, and 3D, respectively). Our results showed that spoligotyping was unable to retrieve five out of the seven main strain lineages as monophyletic groupings (Figure 3B). Even the clear separation between the evolutionary ‘Ancient’ (TbD1+) and ‘Modern’ (TbD1-) clades was missed [31], [32]. It has been argued that even though spoligotyping data may not be ideal for formal phylogenetic analyses, particular “signature” patterns can still be informative for population genetic analyses [26]. For example, the “Beijing” lineage of MTBC has a characteristic loss of 34 spacers (Figure 2), which is caused by a deletion of a genomic region known as RD207 [46]. In other words, this spoligotyping pattern reflects a large sequence polymorphism that is phylogenetically informative (Table S1). Comparison of our MLSA dataset to the corresponding spoligotyping data shows that indeed, many strains can be grouped using such “signature” patterns. However, others cannot be classified properly because their spoligotyping patterns are either ambiguous or uninformative (Figure 2) [47].

**Figure 4:**
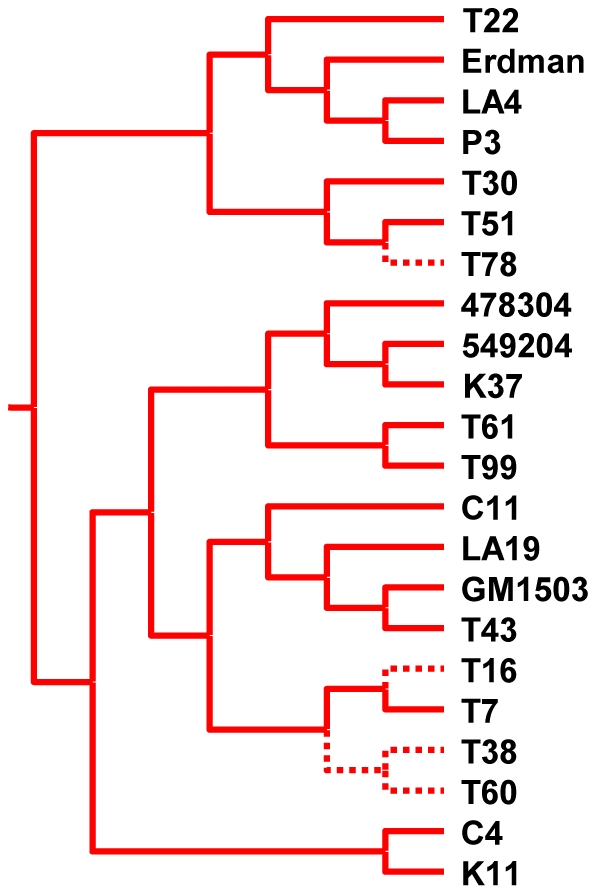
One example of homoplasy in the MIRU-VNTR-based phylogeny for the red strain lineage. The SNP C→G is shared by the strains T60, T38, T16, and T78 (dashed branches). These strains form a monophyletic group in the MLSA phylogeny (Figure 2). By contrast, the MIRU-VNTR-based topology splits these strains into three artificial groups, implying the same C→G change occurred three times independently.

The phylogenetic accuracy of MIRU-VNTR-typing was better than spoligotyping overall, but depended on the number of loci included in the analysis. As observed previously [28], while 15-loci-MIRU-VNTR was prone to phylogenetic misclassification (Figure 3C), 24-loci-MIRU-VNTR was more informative with most strain lineages appearing as monophyletic groups (Figure 3D). However, a closer look revealed several qualitative incongruencies with respect to the MLSA gold standard. At the main lineage level, the green, orange, and blue strains did not appear as monophyletic groupings in the 24-loci-MIRU-VNTR phylogeny (Figure 3D). Furthermore, additional incongruence became evident at the sub-lineage level. To show this, we performed an analysis restricted to strains from the red lineage. We mapped onto the 24-loci-MIRU-VNTR phylogeny all 27 phylogenetically informative SNPs found in red strains based on our MLSA dataset (Figure 2 and Figure 3). We found that 13 of these (48%) were incompatible with the 24-loci-MIRU-VNTR topology (Figure 4 and Figure S2).

A more quantitative way of evaluating the phylogenetic performance of different genotyping methodologies is by comparing the statistical support for each clade. As discussed above, our MLSA-based phylogenies exhibited high statistical support for all strain lineages, irrespective of the phylogenetic method used (Figure 3A, Figure S1). By contrast, for both MIRU-VNTR methodologies bootstrap values were low and thus multiple alternative topologies equally likely (Figure 3C and D). Phylogenetic congruence testing is another quantitative way of comparing phylogenetic topologies. It provides a statistical framework to evaluate how well the MLSA data fits the non-sequence-based phylogenies by calculating a likelihood value associated with each of the methods. Our analysis revealed that the phylogenies derived from spoligotyping and MIRU-VNTR were significantly incompatible with the MLSA data (Table 1). This result is particularly important given that among the various tests available, the Shimodaira-Haegawa test we used here tends to be the most conservative [48].

**Table 1:** Phylogenetic congruence test.

Topology	logL	difference	SH (p-value)
**Spoligotyping**	−95297.8	2826.06	<0.01
**15-loci-MIRU1-VNTR**	−93459.9	988.21	<0.01
**24-loci-MIRU-VNTR**	−93158.4	686.65	<0.01
**MLSA (SNPs)**	−92471.7	0	n.s.

For each topology the likelihood associated to the MLSA alignment and the difference between this value with the highest likelihood is shown (fourth column).

**Figure 5:**
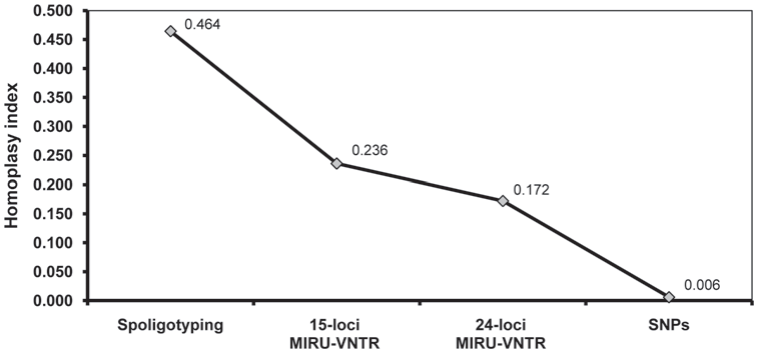
Comparison of the homoplasy index (HI) across the different genotyping methods. HI was calculated based on the number of observed changes at each character compared to the expected number of changes assuming absence of homoplasy. Figure S3 shows several examples of homoplasy for individual MIRU-VNTR loci where the same number of repeats appear in unrelated branches of the tree.

We suspected the reason for the low bootstrap support in the non-sequence-based phylogenies, and the statistically significant incongruence between these phylogenies and the MLSA data was because of homoplasy. Both spoligotyping and MIRU-VNTR are based on a limited number of loci, and the markers used evolve rapidly with a tendency to converge [40]. To test this hypothesis, we calculated the homoplasy index for each marker (Figure 5). As expected based on our qualitative analysis (Figure 3B), the highest homoplasy was found in spoligotyping patterns. Moreover, both the 15-loci-MIRU-VNTR and 24-loci-MIRU-VNTR data sets retained high levels of homoplasy, whereas in the MLSA data, homoplasy was virtually absent (Figure 5). To further explore instances of convergent evolution in MIRU-VNTR, we mapped all VNTR alleles for each MIRU-VNTR locus onto our MLSA phylogeny. We found that 23 out of 24 (96%) loci showed evidence of convergent evolution (Figure S3).

Taken together, our qualitative and quantitative analysis of CRISPR- and VNTR-based genotyping methods revealed that both types of markers are characterized by significant amounts of homoplasy. Hence using these tools to define deep phylogenetic groupings in MTBC or other bacteria, can be misleading [40], [47]. By contrast, DNA sequencing allows to identify true phylogenetic relationships, and to discover SNPs that can be used as powerful genotyping markers (Table S1) [1].

### Discriminatory Power of MIRU-VNTR Markers Vary by Strain Lineage

Even though SNPs are ideal for defining deep phylogenetic groupings, these markers offer insufficient discriminatory power for routine molecular epidemiological investigation in genetically monomorphic bacterial pathogens [1]. CRISPR-, VNTR-, and other related genotyping methods will thus likely remain important genotyping tools for epidemiological purposes. However, because the relative discriminatory power of particular VNTR- loci has been shown to vary depending on the specific strain background [49], we decided to use our MLSA dataset to study this phenomenon in more detail.

**Figure 6:**
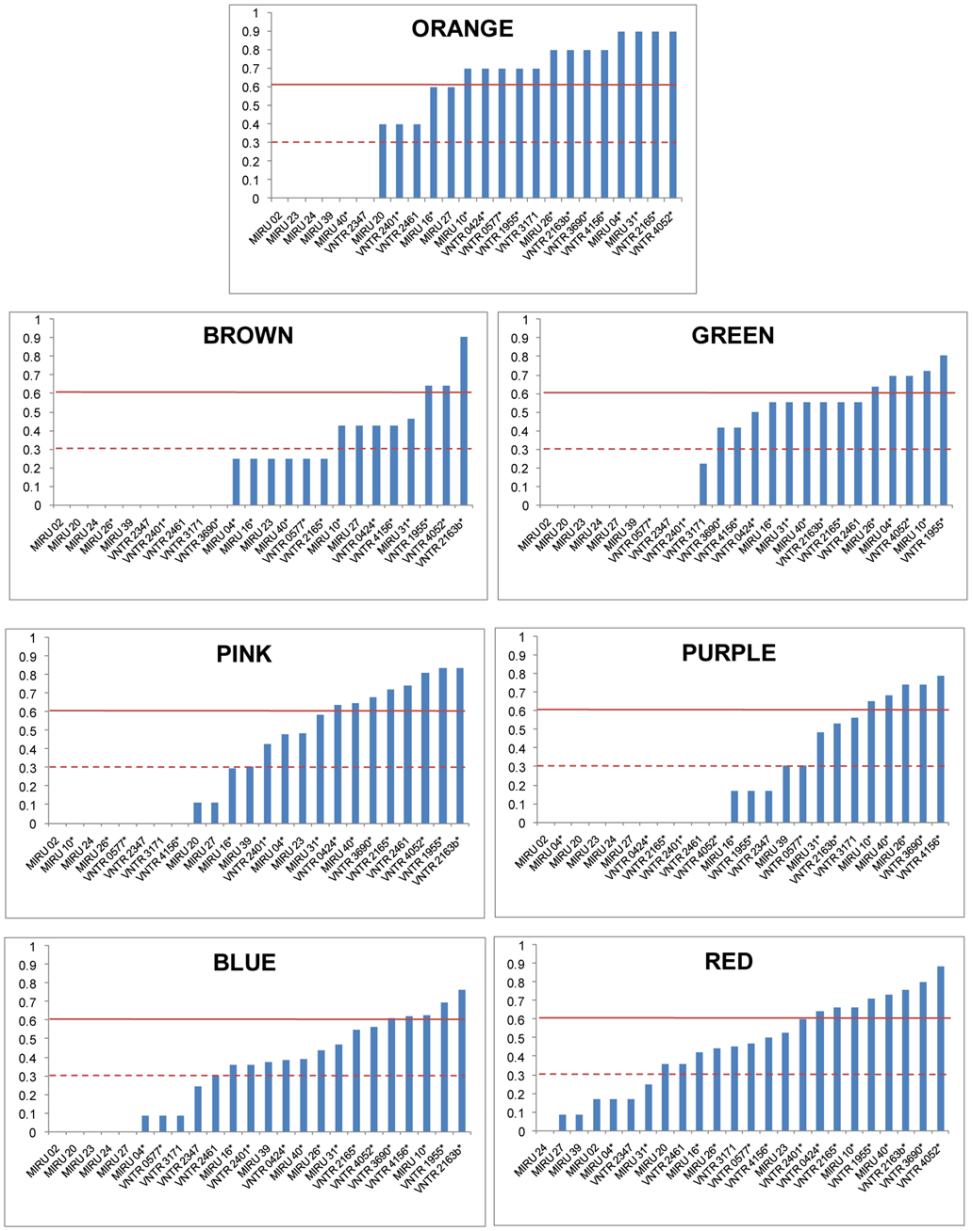
Measure of discriminatory power (HGI) of individual MIRU-VNTR loci by MLSA-defined MTBC strain lineage. Red lines indicate HGI thresholds for highly discriminatory loci (HGI≥0.6, continuous), and intermediate discriminatory loci (HGI≥0.3, dashed), as previously defined [28]. Asterisks indicate MIRU-VNTR loci that have been proposed for standard molecular epidemiological typing of MTBC [28]. See also Table S3.

**Figure 7:**
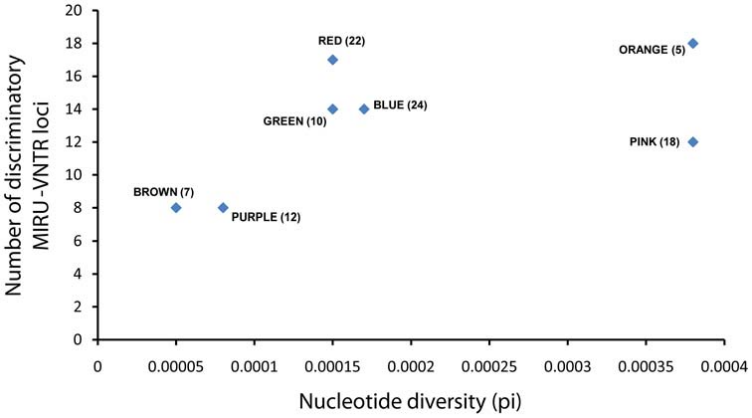
Number of discriminatory MIRU-VNTR loci (HGI≥0.3) as a function of intra-lineage nucleotide diversity (Pi). The number next to the lineage designation indicates the number of strains analyzed for each MTBC lineage.

The discriminatory power of a given genotyping technique can be assessed using the Hunter Gaston Index (HGI) [50]. A high HGI indicates a given molecular marker or methodology is able to correctly classify closely related strains. To test whether the discriminatory power of the standard 24 MIRU-VNTR loci differed by MTBC lineage, we calculated the HGI for each locus separately for each of the main MTBC lineages (Figure 2, Figure 3A). We found that for most strain lineages, the majority of the MIRU-VNTR loci exhibited limited discriminatory power (Figure 6, Table S3). Moreover, the MIRU-VNTR loci that exhibited the highest HGI in one strain lineage were not necessarily the ones with the highest discriminatory power in other strain lineages. The fact MIRU-VNTR loci show the highest discriminatory power for the red lineage suggests that red strains were overrepresented during the original development of this genotyping technique [28]. Some strain lineages in our MLSA dataset were represented by fewer strains than other lineages, which could have influenced our analysis. To test this possibility, we analyzed the intra-lineage nucleotide diversity and compared it to the number of discriminatory loci in each lineage. We found no significant correlation between these two factors (Spearman's rho 0.62, p-value 0.14). Furthermore, in three out of four strain lineages harbouring equal or greater amounts of nucleotide diversity compared to the red lineage, the number of discriminatory loci was lower than in the red lineage (Figure 7).

In sum, our results demonstrate that VNTR loci can exhibit different discriminatory power in different bacterial strain lineages. These findings caution that selection of molecular markers for epidemiological typing should be based on large and globally representative strain collections. Moreover, our findings indicate that to maximize discriminatory power and minimize genotyping costs, only those VNTR markers should be used that offer the highest discriminatory power within a particular strain lineage (Table S3).

## Discussion

Discrimination between strains of pathogenic bacteria is crucial. From an epidemiological perspective, molecular investigation contributes to the control of infectious diseases, both locally and globally. In addition, molecular typing improves our understanding of the basic biology of bacterial pathogens, including differences in virulence and transmissibility, or the variable effectiveness of vaccines and drugs. Unfortunately, the properties of molecular markers required to address both local and global levels of bacterial diversity are unlikely to be met by a single marker [51]. This problem is particularly acute in genetically monomorphic bacteria [1]. Because standard sequence-based genotyping such as MLST are not applicable in these bacteria, non-sequence-based tools, including CRISPR- and VNTR-based techniques, have become the gold standard for routine genotyping of these species. These tools have been applied very successfully to address a variety of epidemiological questions [25].

However, the results presented here argue against the use of these methods for evolutionary studies. The high propensity for convergent evolution and the resulting homoplasies are a significant drawback for defining deep phylogenetic relationships. Although the phylogenetic performance of VNTR-based typing was superior to that of the CRISPR-based method, phylogenies inferred using these markers show little statistical support. Furthermore, both of these typing methods are limited because little information exists with respect to the mode of molecular evolution of the respective molecular markers. This limitation is particularly important for spoligotyping and other CRISPR- based methods where it is virtually impossible to know whether the loss of multiple adjacent sequence spacers is due to a single or multiple evolutionary events. A single-step model of evolution has recently been proposed for VNTR loci in MTBC [30], but more studies are needed to confirm this model. For DNA sequence data on the other hand, multiple models of molecular evolution have been developed based on empirical data, and a robust statistical framework exists to evaluate the validity of these models for inferring phylogenetic relationships [52].

While deep phylogenetic information might be of little relevance for molecular epidemiology, unequivocal classification of bacterial strains is essential for many other applications. For example, elucidating the evolutionary history and global spread of bacterial pathogens requires robust strain assignment [1]. Furthermore, being able to define strains unambiguously is essential if phenotypic associations are to be unveiled. The mere fact that genetically monomorphic bacteria harbour little DNA sequence variation does not necessarily mean all strains of a given species behave the same [53]. In fact in MTBC, there is mounting evidence that strain diversity plays a role in the outcome of TB infection and disease [54], [55], [56], [57]. To detect putative clinical or experimental phenotypes, assignment of individual bacterial strains to specific clades or strain lineages has to rely on phylogenetically well-defined groupings. If bacterial strains are misclassified because of inappropriate genotyping methods, the statistical power to detect true associations will be reduced.

DNA sequencing costs have been decreasing exponentially [58], and full genome sequencing of bacteria has the potential to replace standard bacterial genotyping in the near future [59]. This prospect is particularly relevant for genetically monomorphic pathogens [60]. By interrogating the whole genome, sufficient sequence diversity will be detected to differentiate between individual strains. Furthermore, because of the comprehensive nature of full genome data, they can be used for both fine typing in an epidemiological context and large-scale evolutionary analyses. Several recent reports in *S. typhi* [3], *Brucella* spp. [61], and *Francisella tularensis* [62], have highlighted the potential of comparative whole genome sequencing for elucidating the global population structure of genetically monomorphic bacterial pathogens. However, even though next-generation DNA sequencing is becoming more readily available, such large-scale projects are likely to remain limited to specialized sequencing centers for some time. Until high-throughput genome-sequencing of bacteria becomes more affordable, generating genotyping data for local epidemiology and broader applications in monomorphic microbes will remain challenging. One way to address this challenge is to combine robust lineage-specific markers with highly discriminatory molecular epidemiological typing. Our results demonstrate that CRISPR- and VNTR-based markers can be used for initial exploratory screening of strains. However, because of the inherent phylogenetic limitations of these tools, final strain assignment to specific strain lineages should be based on more robust markers such as SNPs or LSPs.

The data presented here for MTBC suggest an approach, in which the main strain lineages are first identified by SNP-typing. Many SNP-typing technologies have been developed over the years, some of which are more affordable than others [38], [39]. Because lineage-specific SNPs are mutually exclusive in MTBC (Table S1), not all need to be typed in every strain, which can reduce costs. Once the main strain lineages are known, but further molecular epidemiological discrimination is necessary, lineage-specific sets of most discriminatory VNTR markers can be used to separate individual strains within each lineage (Table S3). Such an approach would generate accurate data for epidemiological and evolutionary applications, as well as for classification of strains during clinical or experimental association studies. Similar combined SNP/VNTR-typing schemes could be developed for other genetically monomorphic bacterial pathogens.

## Materials and Methods

### Bacterial Strains and Molecular Typing

The bacterial strains included in this study are representative the global diversity of MTBC as shown previously [31]. MLSA data including 89 genes or 70 kbp per strain was generated by direct DNA sequencing of PCR products as described [31]. Spoligotyping and 15-loci-MIRU-VNTR and 24-loci-MIRU-VNTR genotyping was performed according to internationally standardized protocols [21], [28]. A total of 97 strains which had all genotyping data available was used for the further analyses.

### Data Analysis

To determine the spoligotyping pattern of each strain, each of the 43 spacers was treated as a binary character indicating presence (1) or absence (0). Distances between isolates were calculated using the Jaccard index as implemented in Bionumerics 5.1. A neighbour-joining (NJ) tree was obtained using these distances. For the MIRU-VNTR analysis, Populations 1.3 was used to generate a distance matrix and a phylogenetic tree using the Nei distance [63]. From the distance matrix a NJ tree was obtained and support for each clade was evaluated by generating 1,000 bootstrap pseudoreplicates. For the sequence based phylogenetic inference a concatenate alignment of the 89 genes sequenced by Hershberg et al. [31] was obtained after removing those strains with no MIRU-VNTR or spoligotype information. From the resulting 65,829 base pair alignment we extracted the variable positions (n = 339, Supplementary Table S2) and used them for the phylogenetic analysis. The MLSA tree was obtained by the NJ method, maximum likelihood and Bayesian inference. The NJ analysis was implemented in MEGA 4 [64] using the observed number of changes and 1,000 pseudo-replicated. More complex models were implemented for the maximum-likelihood and Bayesian analyses. We used Modeltest 3.7 [65] to determine the best fit model of nucleotide evolution following the Akaike information criterion [66]. After Modeltest analysis, the TVM model was applied for both analyses. The maximum-likelihood estimation was implemented in PHYML 3.0 [67] without substitution rate heterogeneity correction or invariant estimation as recommended by Modeltest. Clade support was evaluated by analyzing 1,000 bootstrap pseudo-replicates. The Bayesian analysis was run with MrBayes 3.1.2 [68]. This program approximates the posterior probabilities of the phylogenetic tree using a Markov Chain Monte Carlo (MCMC) method. Four chains in two replicates were run during 2 million generations, convergence was evaluated using Tracer 1.4 and accepted when the effective sample sizes of all parameters combining both runs reached 100 as recommended. The final topology and Bayesian *a posteriori* support values for clades were obtained from the consensus tree after discarding the first 10% generations as burn-in.

Based on the high congruence of our LSP and MLSA analyses [31], [33], [34], [35], we assumed that the MLSA topology and lineage classification reflects the true evolutionary history of MTBC. Therefore we tested the specific hypothesis of whether the topologies obtained from the three non-MLSA topologies (spoligotypes, 15-loci-MIRU-VNTR, 24-loci-MIRU-VNTR) were congruent with the MLSA data. The Shimodaira-Hasegawa maximum likelihood test [69] of competing phylogenetic hypothesis was used with 1,000 RELL pseudo-replicates as implemented in Tree-Puzzle [70] to test whether the difference of likelihoods between the best tree and the competing hypotheses were significantly different from zero (alpha at 0.005 after correcting for multiple trees comparisons). The homoplasy index was calculated by fitting the data from each marker to the corresponding topology using PAUP 4.0 b [71]. We used Mesquite 2.6 to map characters across phylogenies. The discriminatory power of each MIRU-VNTR locus was evaluated using the Hunter-Gaston discriminatory index (HGI) [50].

## Supporting Information

Figure S1 Multilocus sequence analysis phylogeny of 97 MTBC strains.
(0.03 MB PDF)

Figure S2 Incongruence of the 24-loci-MIRU-VNTR phylogeny of the strains belonging to the red lineage.
(0.68 MB PDF)

Figure S3 Homoplasy in the MIRU-VNTR loci.
(1.05 MB PDF)

Table S1 Phylogenetically informative SNPs for genoptyping of MTBC.
(0.05 MB XLS)

Table S2 24-loci-MIRU-VNTR, spoligotyping, and SNP data from the 97 MTBC strains included in this study.
(0.58 MB XLS)

Table S3 Discriminatory MIRU-VNTR loci by MTBC lineage.
(0.04 MB XLS)
