# Supplementary material for: Genotyping of Genetically Monomorphic Bacteria: DNA Sequencing in Mycobacterium tuberculosis Highlights the Limitations of Current Methodologies
Source: PLoS One. 2009 Nov 12;4(11):e7815. doi: 10.1371/journal.pone.0007815 (PMC2772813; doi:10.1371/journal.pone.0007815)
Supplement: Figure S1 — Multilocus sequence analysis phylogeny of 97 MTBC strains. (0.03 MB PDF) [file pone.0007815.s001.pdf]

Supplementary Figure 1. Multilocus sequence analysis phylogeny based on 339 SNPs identified in 89 genes of 97 *Mycobacterium tuberculosis* complex strains. The same topology was obtained by neighbour-joining (NJ), maximum likelihood (ML) and Bayesian inference methods (BI) (see main methods for details). Values on the nodes represent percentage of clade support as obtained from 1,000 bootstrap pseudo-replicates for the NJ and ML analyses and Bayesian *a posteriori* values for the BI analysis. Asterisk indicates clade support lower than 50%.

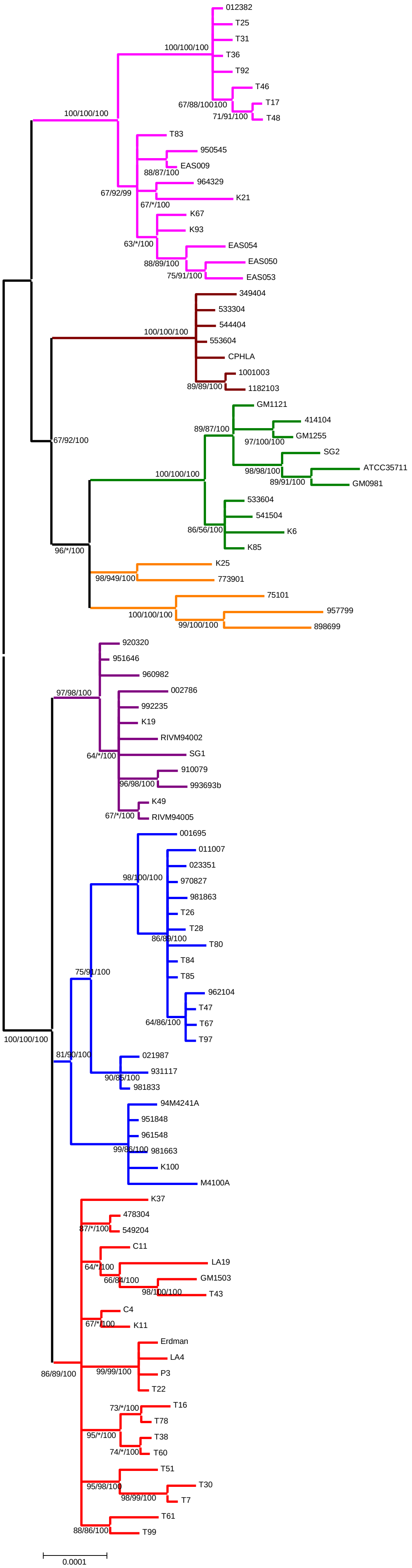
012382
T25
T31
100/100/100
T36
T92
T46
67/88/100100
T17
71/91/100
T48
100/100/100
T83
950545
EAS009
88/87/100
964329
67/92/99
K21
67/*/100
K67
K93
63/*/100
EAS054
EAS050
88/89/100
75/91/100
EAS053
349404
533304
544404
100/100/100
553604
CPHLA
1001003
89/89/100
1182103
GM1121
414104
89/87/100
GM1255
67/92/100
97/100/100
SG2
ATCC35711
98/98/100
89/91/100
GM0981
100/100/100
533604
541504
K6
86/56/100
K85
96/*/100
K25
98/949/100
773901
75101
957799
100/100/100
99/100/100
898699
920320
951646
960982
97/98/100
002786
992235
K19
RIVM94002
SG1
64/*/100
910079
96/98/100
993693b
K49
67/*/100
RIVM94005
001695
011007
023351
98/100/100
970827
981863
T26
T28
86/89/100
T80
T84
T85
75/91/100
962104
T47
T67
64/86/100
T97
100/100/100
81/90/100
021987
931117
90/85/100
981833
94M4241A
951848
961548
99/86/100
981663
K100
M4100A
K37
478304
87/*/100
549204
C11
LA19
64/*/100
66/84/100
GM1503
98/100/100
T43
C4
67/*/100
K11
Erdman
LA4
86/89/100
99/99/100
P3
T22
73/*/100
T16
T78
95/*/100
T38
74/*/100
T60
T51
95/98/100
T30
98/99/100
T7
T61
88/86/100
T99
0.0001
