# Supplementary material for: Genotyping of Genetically Monomorphic Bacteria: DNA Sequencing in Mycobacterium tuberculosis Highlights the Limitations of Current Methodologies
Source: PLoS One. 2009 Nov 12;4(11):e7815. doi: 10.1371/journal.pone.0007815 (PMC2772813; doi:10.1371/journal.pone.0007815)
Supplement: Figure S2 — Incongruence of the 24-loci-MIRU-VNTR phylogeny of the strains belonging to the red lineage. (0.68 MB PDF) [file pone.0007815.s002.pdf]

Supplementary Figure 2. Incongruence of the 24-loci-MIRU-VNTR phylogeny of the strains belonging to the red lineage. Our multilocus sequence analysis identified 27 phylogenetically informative SNPs in the red lineage. When mapping each individual SNPs onto the 24-loci-MIRU-VNTR phylogeny, 13 of these 27 SNPs suggested convergent evolution (indicated in separate Figure panels below) which would lead to high degrees of homoplasy in the MLSA data. However, phylogenetic analyses of our MLSA data clearly demonstrate that these sequence data harbour negligible amounts of homoplasy (Figure 5).

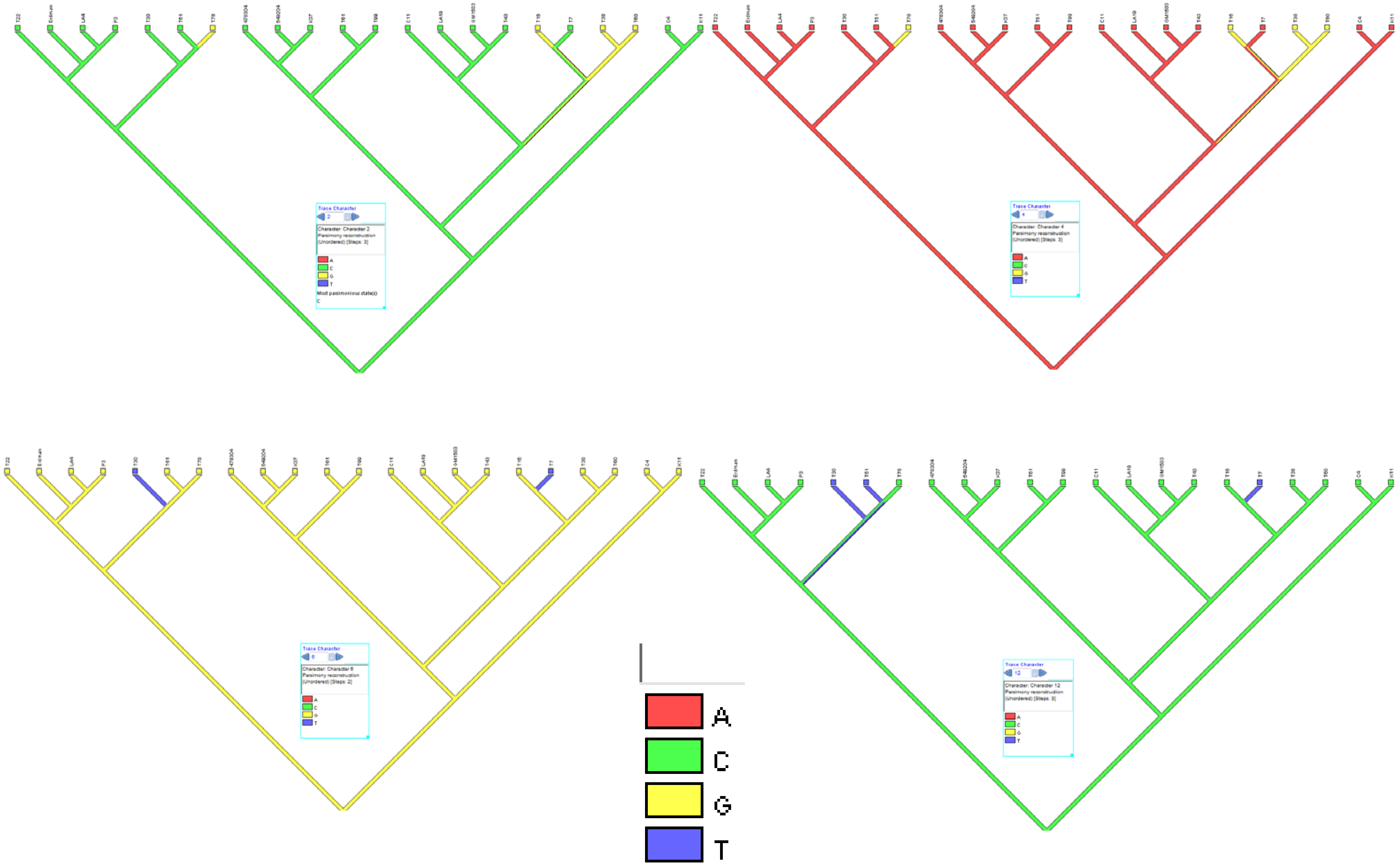
A
C
G
T

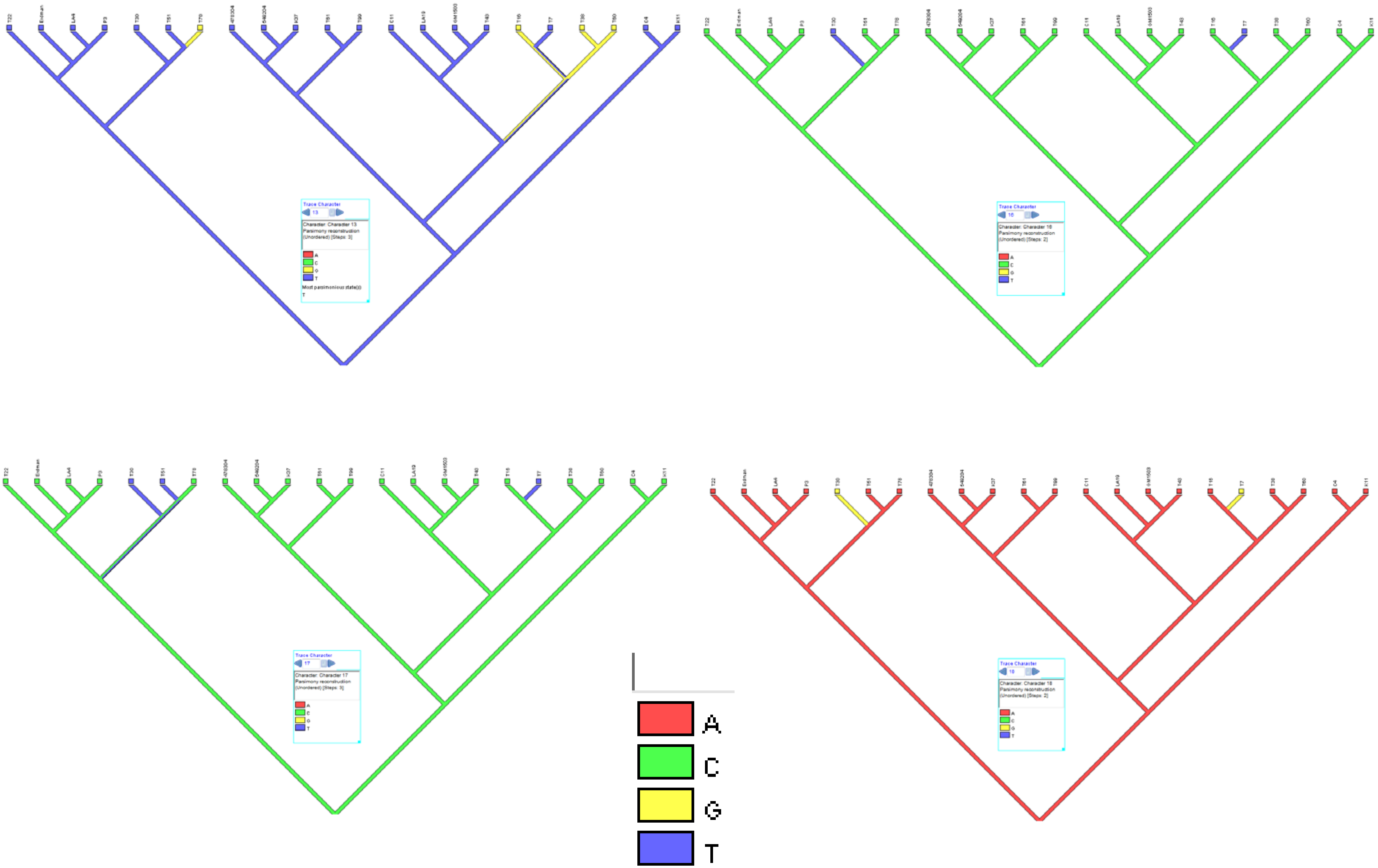
A
C
G
T

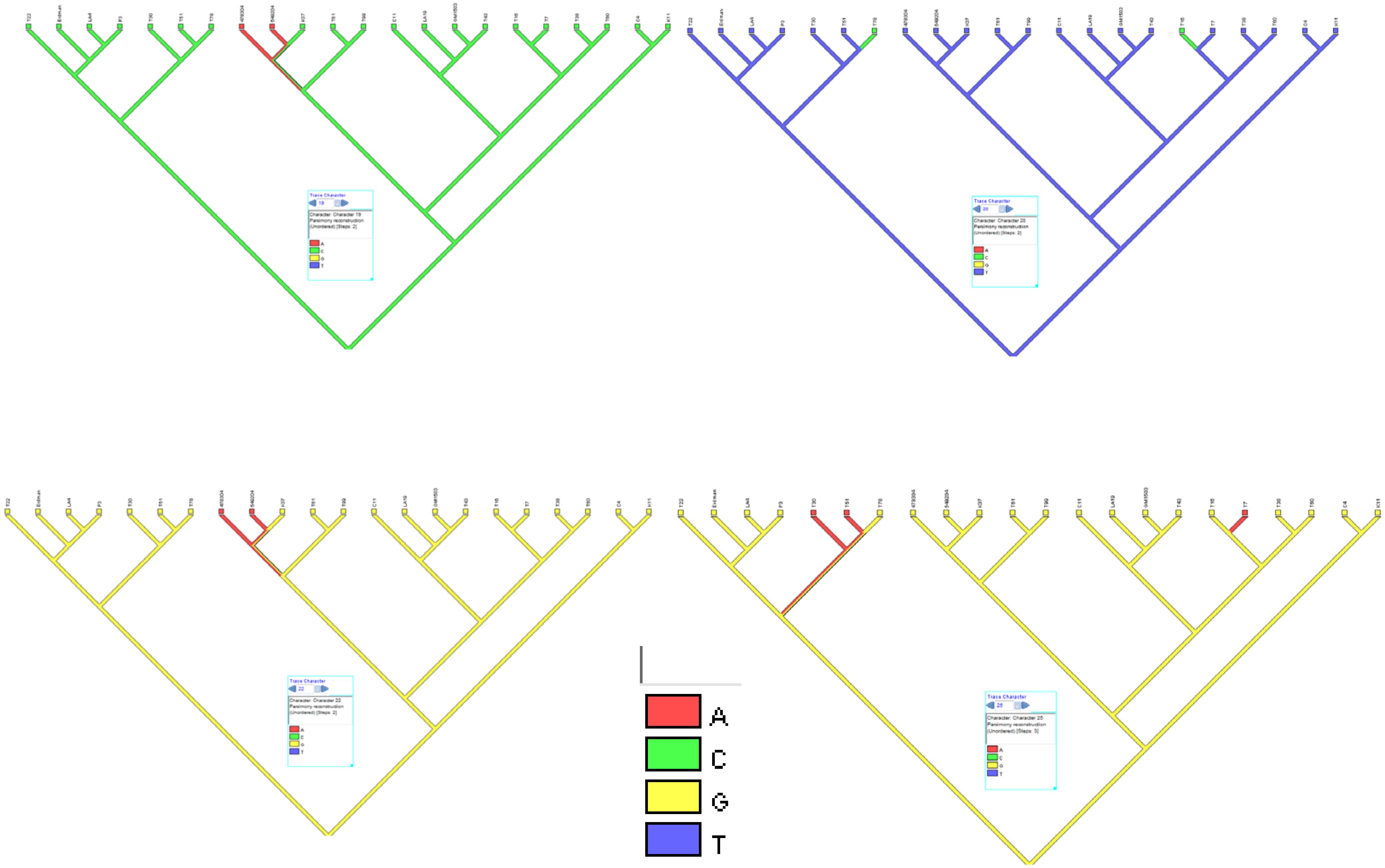
A
C
G
T

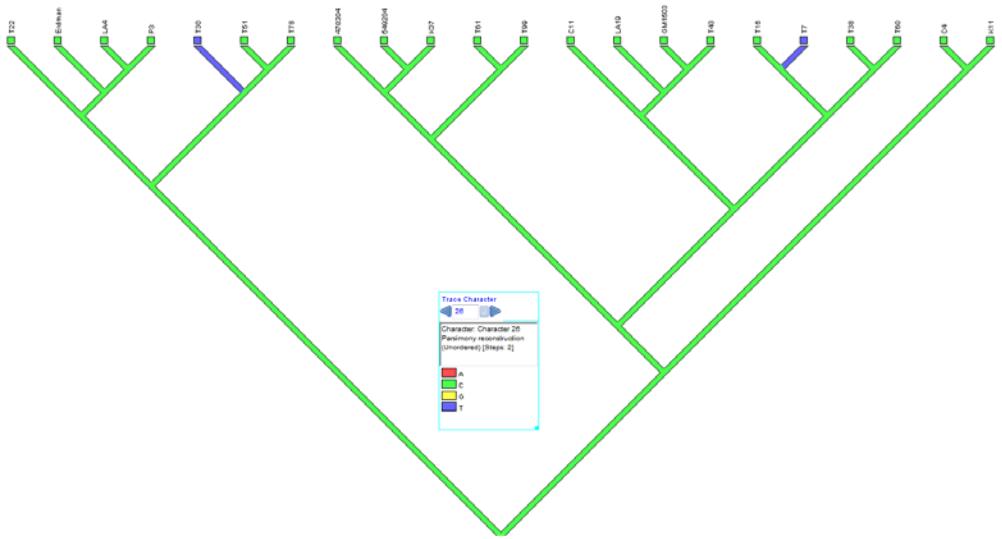
T22
Erdman
LA4
P3
T30
T51
T61
C11
LA19
GM1503
T7
C4
Trace Character
26
Character: Character 26
Parsimony reconstruction
(Unordered) [Steps: 2]
A
C
G
T

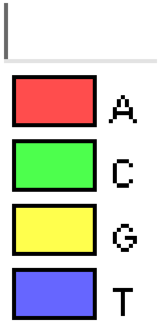
A
C
G
T
