# Supplementary material for: Genotyping of Genetically Monomorphic Bacteria: DNA Sequencing in Mycobacterium tuberculosis Highlights the Limitations of Current Methodologies
Source: PLoS One. 2009 Nov 12;4(11):e7815. doi: 10.1371/journal.pone.0007815 (PMC2772813; doi:10.1371/journal.pone.0007815)
Supplement: Figure S3 — Homoplasy in the MIRU-VNTR loci. (1.05 MB PDF) [file pone.0007815.s003.pdf]

Supplementary Figure 3. Homoplasy in the MIRU-VNTR loci. For each of the 24 standard MIRU-VNTR loci, the different allele variants were mapped onto the MLSA phylogeny (illustrated in separate Figure panels below). Each allele variant is represented by a different colour. 23 out of 24 loci showed clear evidence of convergent evolution.

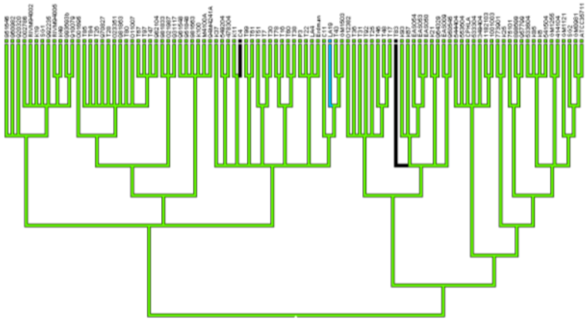

MIRU02

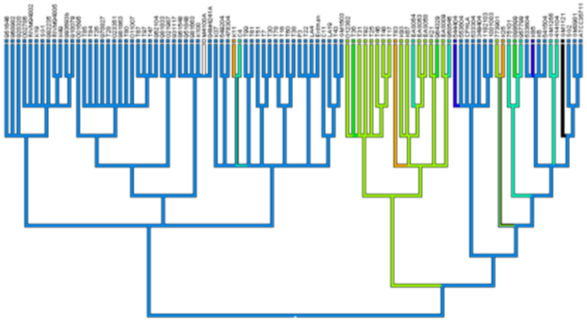

MIRU04

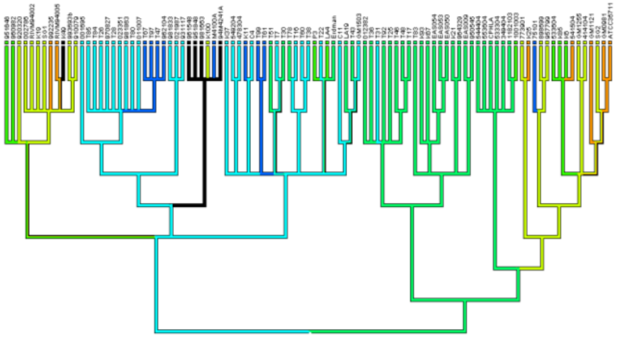

MIRU10

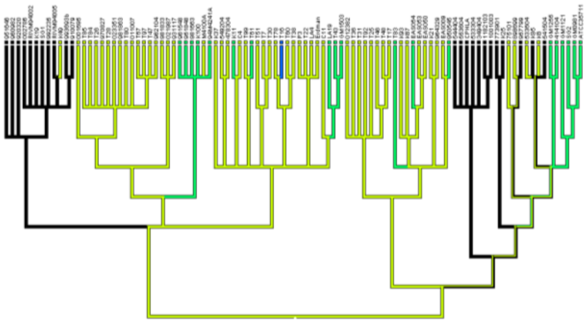

MIRU16

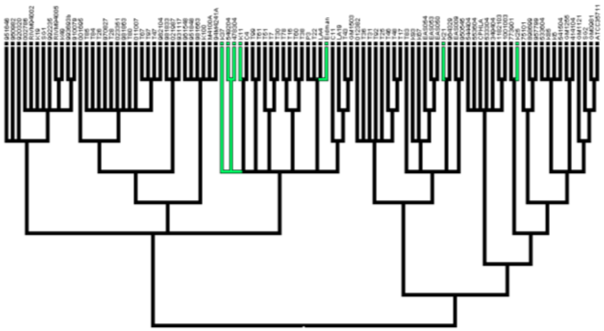

MIRU20

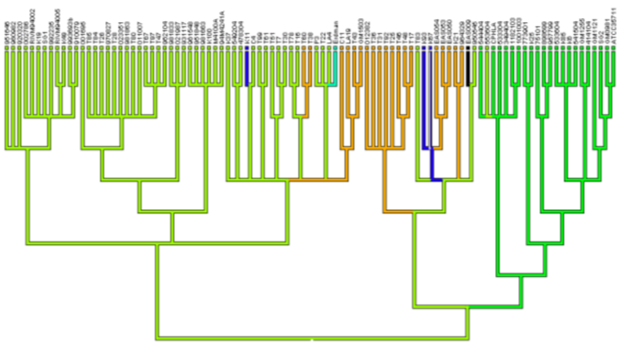

MIRU23

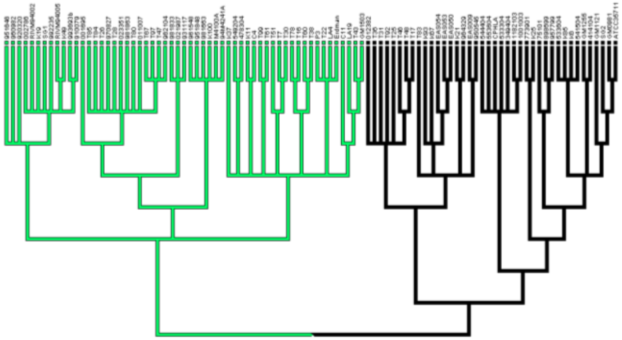

MIRU24

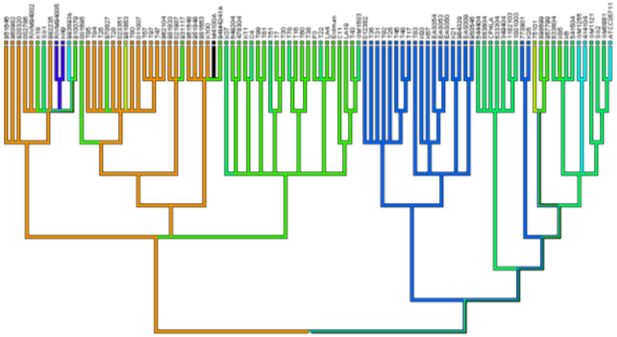

MIRU26

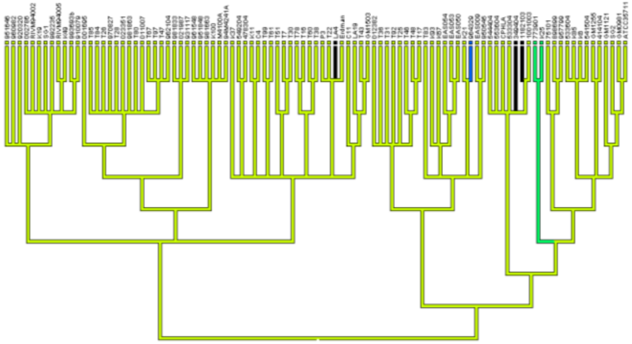

MIRU27

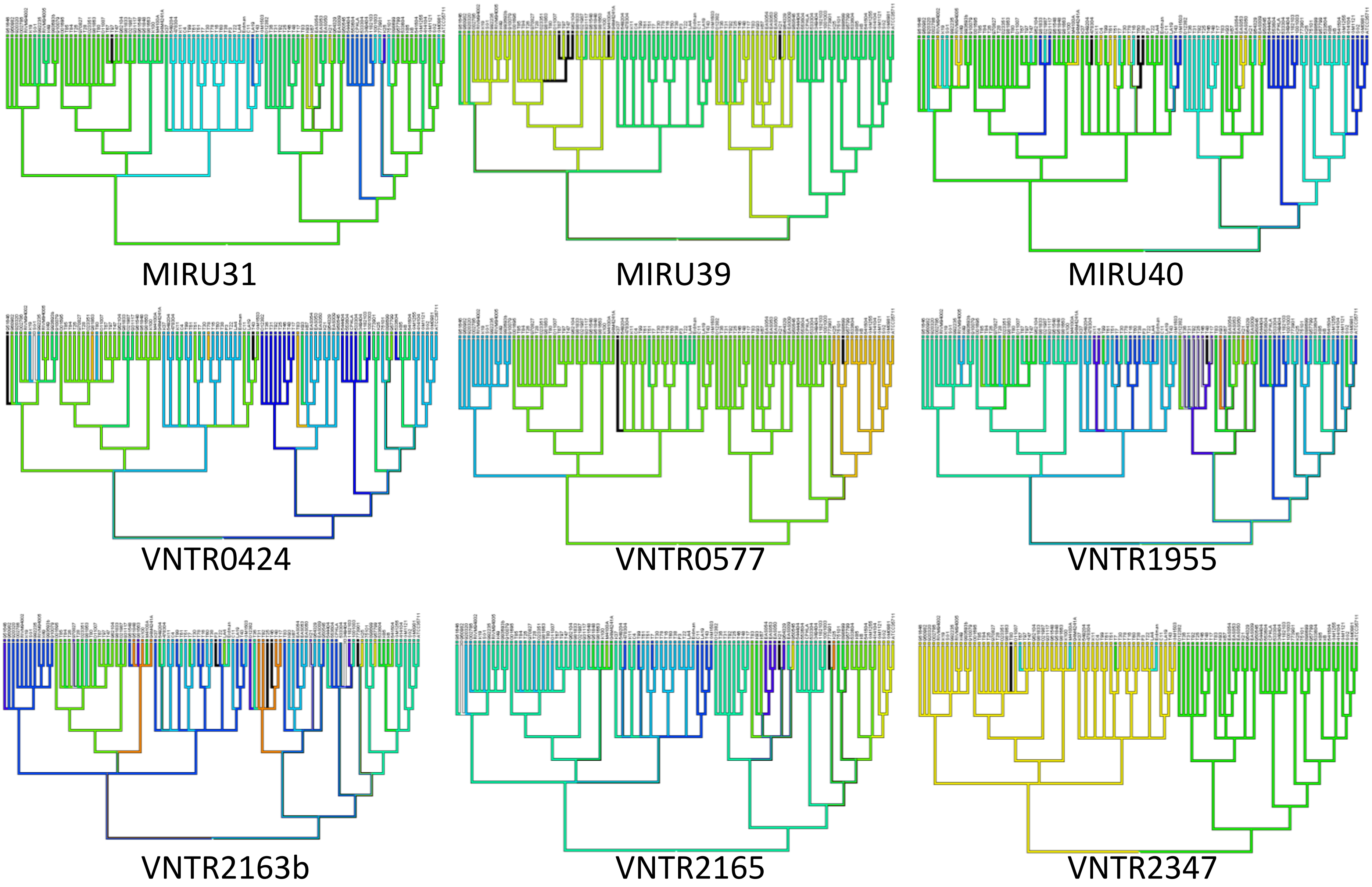

MIRU31
MIRU39
MIRU40
VNTR0424
VNTR0577
VNTR1955
VNTR2163b
VNTR2165
VNTR2347

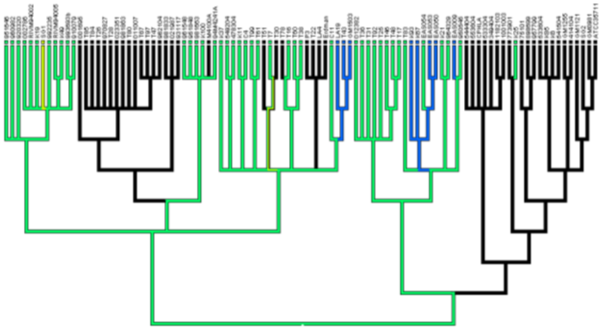

VNTR2401

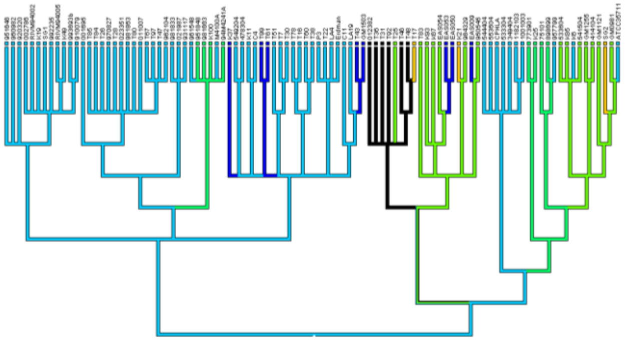

VNTR2461

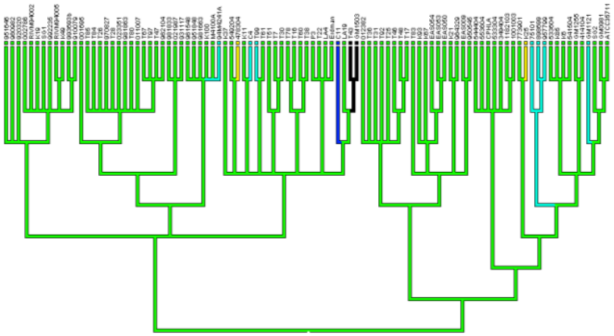

VNTR3171

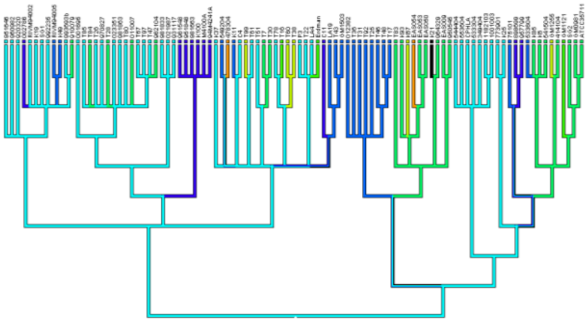

VNTR3690

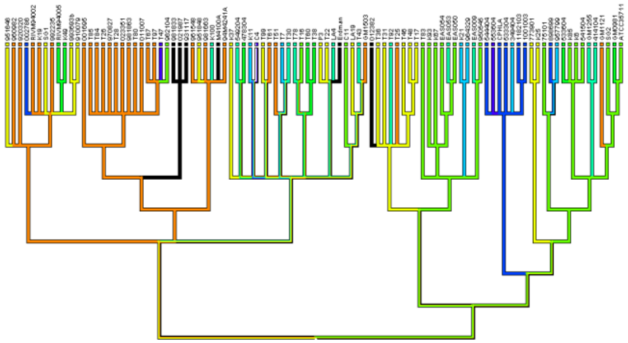

VNTR4052

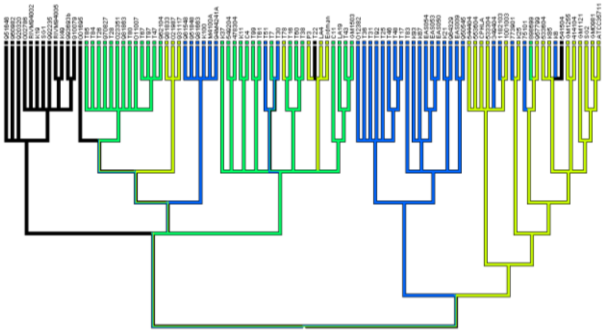

VNTR4156

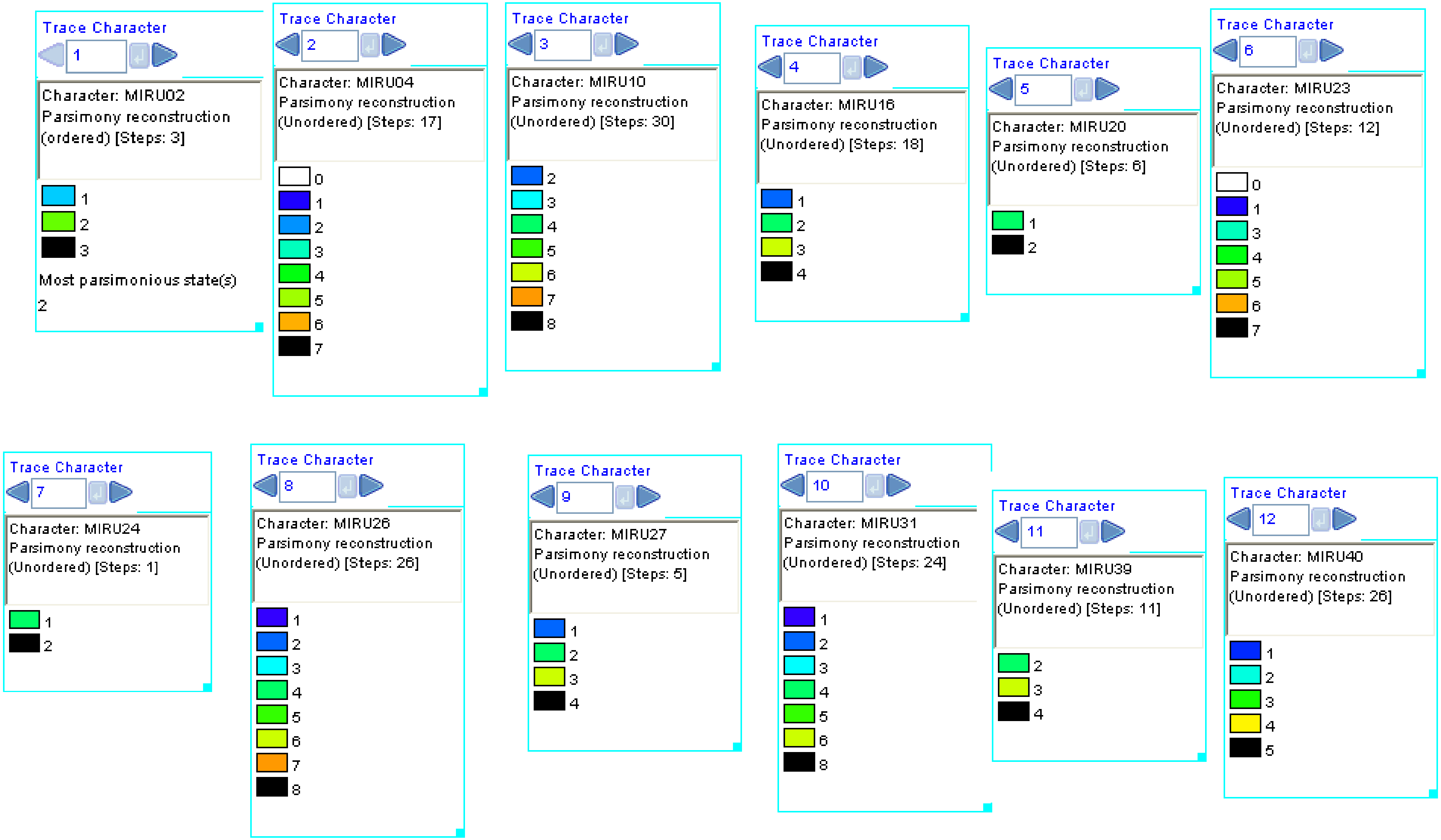
Trace Character
1
Character: MIRU02
Parsimony reconstruction (ordered) [Steps: 3]
1
2
3
Most parsimonious state(s)
2
Trace Character
2
Character: MIRU04
Parsimony reconstruction (Unordered) [Steps: 17]
0
1
2
3
4
5
6
7
Trace Character
3
Character: MIRU10
Parsimony reconstruction (Unordered) [Steps: 30]
2
3
4
5
6
7
8
Trace Character
4
Character: MIRU16
Parsimony reconstruction (Unordered) [Steps: 18]
1
2
3
4
Trace Character
5
Character: MIRU20
Parsimony reconstruction (Unordered) [Steps: 6]
1
2
Trace Character
6
Character: MIRU23
Parsimony reconstruction (Unordered) [Steps: 12]
0
1
3
4
5
6
7
Trace Character
7
Character: MIRU24
Parsimony reconstruction (Unordered) [Steps: 1]
1
2
Trace Character
8
Character: MIRU26
Parsimony reconstruction (Unordered) [Steps: 26]
1
2
3
4
5
6
7
8
Trace Character
9
Character: MIRU27
Parsimony reconstruction (Unordered) [Steps: 5]
1
2
3
4
Trace Character
10
Character: MIRU31
Parsimony reconstruction (Unordered) [Steps: 24]
1
2
3
4
5
6
8
Trace Character
11
Character: MIRU39
Parsimony reconstruction (Unordered) [Steps: 11]
2
3
4
Trace Character
12
Character: MIRU40
Parsimony reconstruction (Unordered) [Steps: 26]
1
2
3
4
5

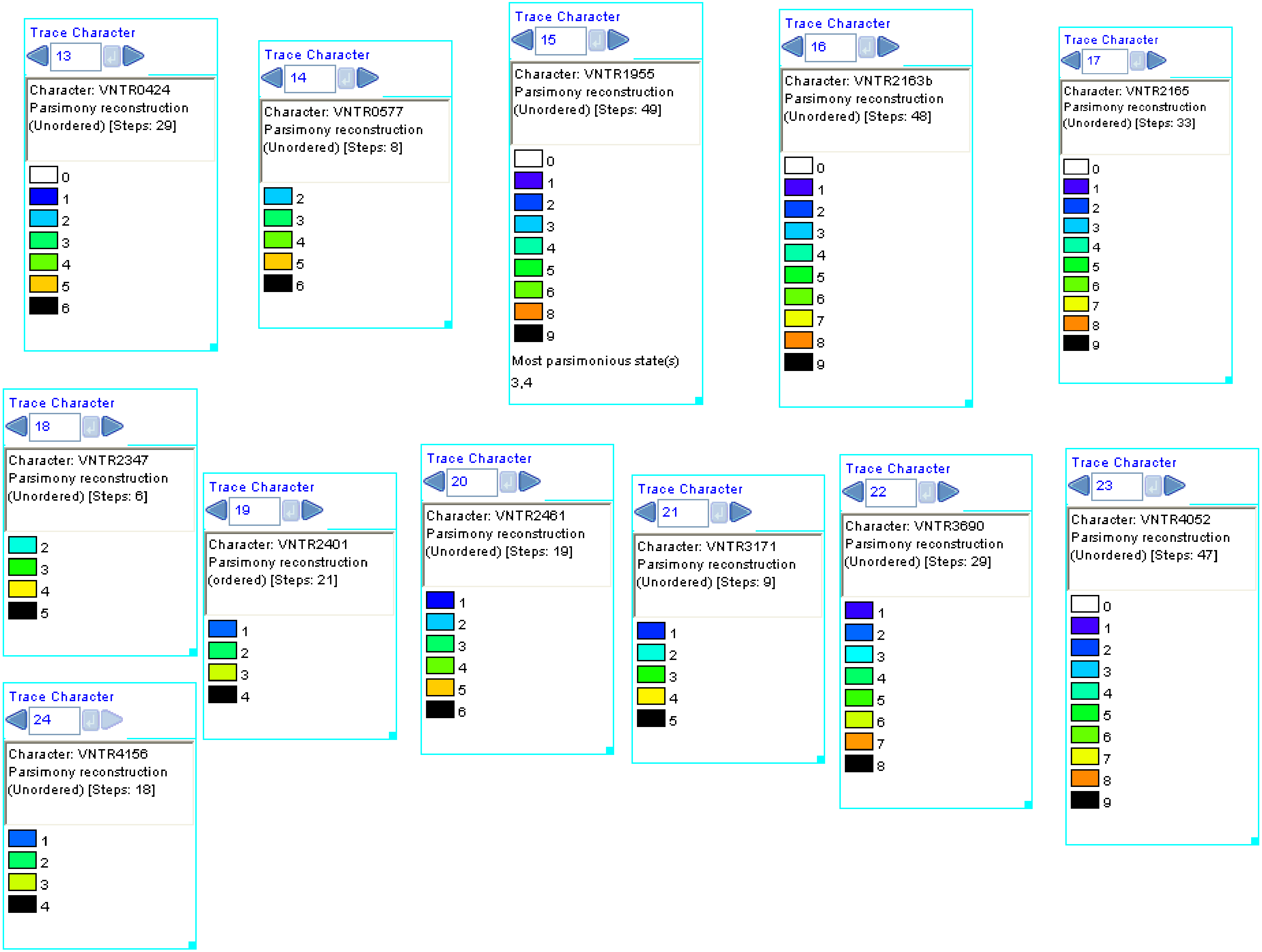
Trace Character
13
Character: VNTR0424
Parsimony reconstruction
(Unordered) [Steps: 29]
0 1 2 3 4 5 6
Trace Character
14
Character: VNTR0577
Parsimony reconstruction
(Unordered) [Steps: 8]
2 3 4 5 6
Trace Character
15
Character: VNTR1955
Parsimony reconstruction
(Unordered) [Steps: 49]
0 1 2 3 4 5 6 8 9
Most parsimonious state(s)
3,4
Trace Character
16
Character: VNTR2163b
Parsimony reconstruction
(Unordered) [Steps: 48]
0 1 2 3 4 5 6 7 8 9
Trace Character
17
Character: VNTR2165
Parsimony reconstruction
(Unordered) [Steps: 33]
0 1 2 3 4 5 6 7 8 9
Trace Character
18
Character: VNTR2347
Parsimony reconstruction
(Unordered) [Steps: 6]
2 3 4 5
Trace Character
19
Character: VNTR2401
Parsimony reconstruction
(ordered) [Steps: 21]
1 2 3 4
Trace Character
20
Character: VNTR2461
Parsimony reconstruction
(Unordered) [Steps: 19]
1 2 3 4 5 6
Trace Character
21
Character: VNTR3171
Parsimony reconstruction
(Unordered) [Steps: 9]
1 2 3 4 5
Trace Character
22
Character: VNTR3690
Parsimony reconstruction
(Unordered) [Steps: 29]
1 2 3 4 5 6 7 8
Trace Character
23
Character: VNTR4052
Parsimony reconstruction
(Unordered) [Steps: 47]
0 1 2 3 4 5 6 7 8 9
Trace Character
24
Character: VNTR4156
Parsimony reconstruction
(Unordered) [Steps: 18]
1 2 3 4
